# Activation of Glutamate Transporter-1 (GLT-1) Confers Sex-Dependent Neuroprotection in Brain Ischemia

**DOI:** 10.3390/brainsci11010076

**Published:** 2021-01-08

**Authors:** Flavia A. Tejeda-Bayron, David E. Rivera-Aponte, Christian J. Malpica-Nieves, Gerónimo Maldonado-Martínez, Héctor M. Maldonado, Serguei N. Skatchkov, Misty J. Eaton

**Affiliations:** 1 Department of Biochemistry, School of Medicine, Universidad Central del Caribe, Bayamón, PR 00960-6032, USA; 415ftejeda@uccaribe.edu (F.A.T.-B.); david.rivera@uccaribe.edu (D.E.R.-A.); 415cmalpica@uccaribe.edu (C.J.M.-N.); 2 School of Chiropractic, Universidad Central del Caribe, Bayamón, PR 00960-6032, USA; geronimo.maldonado@gmail.com; 3 Biology Department, University of Puerto Rico—Río Piedras Campus, Río Piedras, PR 00924-2537, USA; 4 Department of Pharmacology, School of Medicine, Universidad Central del Caribe, Bayamón, PR 00960-6032, USA; hmaldonado1@gmail.com; 5 Department of Physiology and Biochemistry, School of Medicine, Universidad Central del Caribe, Bayamón, PR 00960-6032, USA; serguei.skatchkov@uccaribe.edu

**Keywords:** glutamate, glial cells, brain ischemia, cerebrovascular disease/stroke, sex-specific, glutamate transporters

## Abstract

Stroke is one of the leading causes of long-term disability. During ischemic stroke, glutamate is released, reuptake processes are impaired, and glutamate promotes excitotoxic neuronal death. Astrocytic glutamate transporter 1 (GLT-1) is the major transporter responsible for removing excess glutamate from the extracellular space. A translational activator of GLT-1, LDN/OSU 0212320 (LDN) has been previously developed with beneficial outcomes in epileptic animal models but has never been tested as a potential therapeutic for ischemic strokes. The present study evaluated the effects of LDN on stroke-associated brain injury. Male and female mice received LDN or vehicle 24 h before or 2 h after focal ischemia was induced in the sensorimotor cortex. Sensorimotor performance was determined using the Rung Ladder Walk and infarct area was assessed using triphenyltetrazolium chloride staining. Males treated with LDN exhibited upregulated GLT-1 protein levels, significantly smaller infarct size, and displayed better sensorimotor performance in comparison to those treated with vehicle only. In contrast, there was no upregulation of GLT-1 protein levels and no difference in infarct size or sensorimotor performance between vehicle- and LDN-treated females. Taken together, our results indicate that the GLT-1 translational activator LDN improved stroke outcomes in young adult male, but not female mice.

## 1. Introduction

Stroke is the fifth-leading cause of death in the United States and ranks as one of the leading causes of long-term disability [1]. Ischemic stroke results when an arterial blockage causes interruption of the flow of nutrients and oxygen to an area of the brain. Cells fail to generate sufficient ATP, resulting in energy failure that leads to loss of ionic gradients and dysregulation of neurotransmitter release [2]. In particular, glutamate is released, reuptake processes are impaired, and glutamate binds to its postsynaptic receptors and promotes excitotoxic neuronal death after an ischemic insult [3].

Astrocytes in the Central Nervous System (CNS) are responsible for removing excess glutamate from the extracellular space via the EAAT2 (GLT-1) glutamate transporter [4,5,6]. At first, and for many years, astrocytes were considered support cells with limited structural roles to the neurons. During the last decades, the wide range of roles astrocytes play has been discovered in the brain, including but not limited to homeostasis, ion buffering, and uptake and synthesis of neurotransmitters [7,8,9,10].

Excitotoxicity due to glutamate in the extracellular space is one of the mediators of neuronal death during and after an ischemic insult [3,11,12,13]. During cerebral ischemia, there is a redistribution of ions (K^+^ efflux and influx of Na^+^, Cl^−^ and Ca^2+^) and membrane depolarization of neurons, which results in excessive release of neurotransmitters, particularly glutamate [14]. Glutamate is the principal excitatory neurotransmitter in the mature CNS. During an ischemic insult, increased release of glutamate in conjunction with impaired uptake results in a toxic buildup of extracellular glutamate, leading to overstimulation of ionotropic glutamate receptors and consequent neuronal cell death [15]. It has been shown that 5 min of experimental ischemia can produce an eight-fold increase in extracellular [glutamate] in vivo [16].

The GLT-1 glutamate transporter is a transmembrane protein located in the membrane of astrocytes. It accounts for over 90% of the glutamate uptake into astroglial cells and helps in maintaining healthy levels of glutamate in the extracellular space and limiting the excitotoxicity of glutamate in the CNS [5,6]. Over the last years, GLT-1 has been studied as a potential therapeutic target for neuropathologies, since astrocytes are better preserved than neurons during and after stroke [17]. Studies performed in young, male rats have shown beneficial outcomes in Middle Cerebral Artery occlusion (MCAo) models of ischemic stroke in which infarct size has been reduced by inhibiting the release of excitatory amino acids into the extracellular space. Upregulation of GLT-1 has been shown to decrease lesion sizes in young male rats subjected to focal and global overexpression of GLT-1 by both adeno-associated-viral vector GLT-1 expression [18] and transcriptional activation with the beta-lactam antibiotic, ceftriaxone [19].

Consequent to years of clinical trials, the only non-invasive approved treatment to have shown protective efficacy is thrombolysis via tissue plasminogen activator (tPA), which has to be administered within a 3-to-5-h window after an ischemic insult [20]. In 2014, Kong and colleagues characterized a compound, LDN-OSU 0212023 (LDN), that upregulated GLT-1 protein levels at the post-transcriptional level as fast as two hours after injection. This fast and efficient upregulation in GLT-1 has provided beneficial outcomes in both Amyotrophic Lateral Sclerosis and epilepsy models [21]. This study aims to evaluate the effects of upregulating GLT-1 with LDN as a potential therapeutic for ischemic stroke. We found that post-transcriptional upregulation of GLT-1 with LDN correlates with decreases in infarct size and improvements in sensorimotor performance after focal ischemia in young adult male mice. Furthermore, LDN was effective in improving acute stroke outcomes in male mice when administered prior to or after focal ischemia. In contrast and intriguingly, LDN did not increase GLT-1 levels in young adult female mice nor did it improve stroke outcomes in these female mice.

## 2. Materials and Methods

### 2.1. Animals

**Figure 1:**
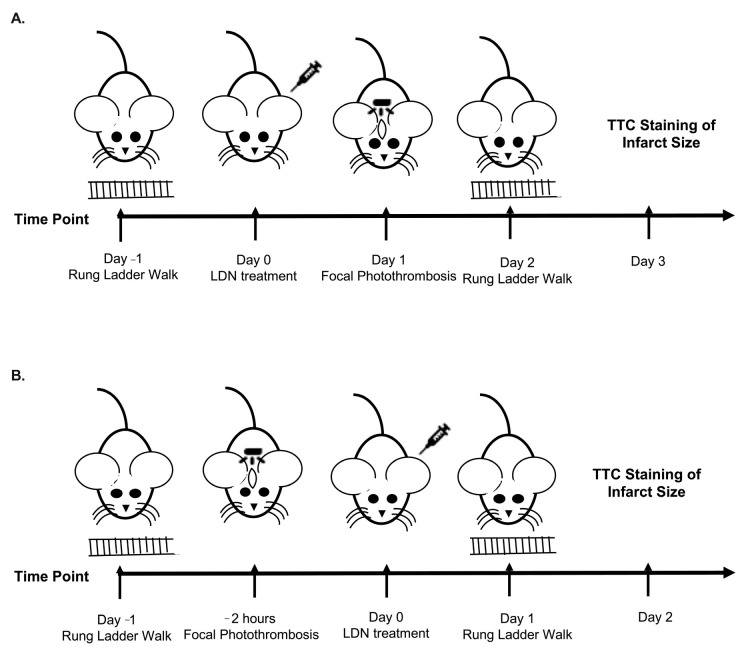
Schematics of Experimental Design. (**A**) Design for experiments administering LDN/OSU 0212320 (LDN) 24 h before focal ischemia. (**B**) Design for experiments administering LDN 2 h after focal ischemia.

Male and Female C57BL/6NCrl mice were purchased from Charles River Laboratories and a breeding colony was established in the Animal Resources Center of Universidad Central del Caribe. Mice were maintained on a 12-h light/dark schedule and had access to food and water ad libitum. In the studies described here, we used 106 animals, 50 males and 56 females, at an age of 10–12 weeks (see Figure 1 for description of Experimental Protocol). This study was performed following NIH Guidelines for the care and use of laboratory animals.

### 2.2. Drugs

Animals were injected with a solution of either 500 µL of vehicle (1% DMSO, 1% polyethylene glycol 400, 0.2% Tween 80, 10% hydroxypropyl-beta-cyclodextrin, and saline) or 40–100 mg/kg LDN/OSU 0212320 (350–500 µL; Aobious; Hopkinton, MA, USA).

### 2.3. Focal Photothrombosis

Unilateral ischemic strokes were induced in the sensorimotor cortex using the photothrombotic method via Rose Bengal injection followed by illumination [22,23]. The mouse was induced to anesthesia with 3.5% of inhaled isoflurane and maintained with 1.5% during the entire procedure. The head was immobilized in a stereotaxic device with a nose cone mask suitable for use with the stereotaxic device. The head was shaved, and a midline incision was made, the skull was cleaned and with a 5µL Hamilton fixed syringe (Hamilton Co. cat no. 80016), the area to be illuminated over the sensorimotor cortex (anteroposterior (A/P), 0.0; mediolateral (M/L), +1.75), was marked [24,25]. The mice were injected i.p. with 10 µL/g body weight with a solution of 7.5 mg Rose Bengal (Sigma-Aldrich, St. Louis, MO, USA) in 1 mL PBS followed by a 5 min period for the dye to diffuse into the blood stream. We built a 1.61 cm^2^ square sheet from marine-grade vinyl containing a 2 mm pinhole and with a thickness of 1 mm. This was positioned over the skull in the region of the sensorimotor cortex. Photothrombosis was then induced by restricted illumination of the marked area using an X-Cite series 120 metal halide lamp (Excelitas Technologies, Waltham, MA, USA) [26]. The light was centered over the pinhole region of the marine-grade vinyl sheet and illuminated for 40 s. After 40 s of illumination at 12.5% power (9.613 mW/cm^2^), the light was turned off and the wound was sutured. During the procedure animal temperature as well as respiration were closely monitored. Animals were observed until they fully recovered from anesthesia, upon which time they were returned to the animal room and subsequently monitored twice a day for signs of distress. Sham mice were subjected to the whole surgical paradigm including the injection of Rose Bengal, but not receive the illumination of the skull with the light. At the time of surgery, animals were not identified as to which were treated with LDN or vehicle, blinding the person in charge of the surgery to the treatments.

### 2.4. Rung Ladder Walk

The rung ladder walk behavioral test was performed to assess sensorimotor deficits in mice receiving photothrombotic lesions, following the methods described in Hines and Haydon [27] and Farr et al. [28] The rung walk apparatus is composed of two Plexiglas walls (69.5 × 15 cm) [27]. Each wall contains 121 holes, 0.20 cm in diameter, spaced 0.5 cm apart, and located 1 cm from the bottom edge of the wall. The holes are filled with 8-cm-long metal bars, with a diameter of 0.10 cm. The walls have 5 cm space apart to allow the passage of a mouse but to prevent it from turning around. The entire apparatus is placed on two standard mouse cages. The first cage served as a neutral start location, and the cage at the exit was the animal’s home cage. Training and presurgery control value experiments were performed 24 h before the photothrombosis with the use of regular patterns which means all rods were set in the rung ladder. 24 h after photothrombosis surgery, mice were then tested with the rung ladder set with an alternating pattern which means that a space was left between rods by removing one rod between two rods. Performance on this test was measured on a scale of 0–6 for each step taken, with a score of 0 being a complete miss of the rung, a score of 1 a deep slip, a score of 2 a slight slip, a score of 3 a replacement, a score of 4 a correction, a score of 5 a partial placement, and a score of 6 being a perfect step [27,29]. Animals were randomly assigned to treatment groups and the animals were color coded to blind the investigator to the treatment group when analyzing the data. We performed preliminary studies in sham animals to ensure that mice receiving focal photothrombosis had behavioral deficits when compared with the sham animals. We tested three sham male and three sham female mice. Sham animals displayed no infarct and had better motor performance than vehicle-treated mice receiving focal ischemic lesions.

### 2.5. Determination of Infarct Volume

Two days after surgery, animals were decapitated, and their brains removed. The cerebellum regions were collected for subsequent quantification of GLT-1 protein levels (see below) whereas the remaining brain including the sensorimotor cortex was cut in 1.0 mm coronal sections using a mouse brain matrix. The sections were stained with triphenyltetrazolium chloride (TTC) (Sigma-Aldrich) (5% TTC in PBS, pH 7.4 for 15–20 min) and photographed. TTC is a salt that can be reduced by metabolically active mitochondria through dehydrogenases. The presence of dehydrogenase activity in the tissue turns the colorless solution into a red solution. The infarcts are metabolically inactive tissue and are denoted by the lack of red color (absence of color) in the brain sections [30]. The area of the infarct was measured with ImageJ Software (NIH; Bethesda, MD, USA) with a photograph of the brain slices taken by a camera positioned at the same height above the brain slices and using a 1 cm square to adjust for minor height differences between photographs. Afterwards, the volume of the total infarct size was calculated using the Sorant formula [31] as described by Hao et al. (2008) [32]. As noted above, the animals were color coded to blind the investigator to the treatment group when analyzing the data.

### 2.6. Determination of GLT-1 Protein Levels by Western Blotting

Cerebellar (collected above) or cortical brain regions were homogenized in lysis buffer containing 10 mmol/L Tris-HCl (pH 8.0), 1 mmol/L EDTA, 0.5 mmol/L EGTA, 1% Triton X-100, 0.1% sodium deoxycholate, 0.1% SDS, 140 mmol/L NaCl, 1 mmol/L of an additional mixture of peptide inhibitors (leupeptin, bestatin, pepstatin, and aprotinin) and 1 mmol/L of Phenylmethylsulfonyl fluoride (PMSF). The samples were manually homogenized using mortars and sonicated for 30 s. The homogenates were then centrifuged at 14,000 *g* for 10 min at 4 degrees C and the supernantants analyzed. Total protein concentrations of supernatants were determined with the Bradford assay (Bio-Rad, Hercules, CA, USA), followed by addition of an appropriate volume of Urea sample buffer (4% SDS, 8 mol/L Urea, 62 mmol/L Tris-HCl, 5% β-Mercaptoethanol, 0.15% Bromophenol Blue and 20 mmol/L EDTA; pH 6.8) to load 10 μg/uL of protein per lane. The samples were electrophoresed on a 4–15% SDS-polyacrylamide gradient gel (Bio-Rad, 4561080) and transferred to polyvinylidene difluoride (PVDF) membranes (Bio-Rad, 1620177). Electrophoresis was performed using a running buffer comprised of 25 mmol/L Tris, 192 mmol/L glycine, 0.1% SDS, pH 8.3. PVDF membranes were immediately washed with PBS-0.4% Tween, followed by 1-h staining with India ink solution (0.1% India ink in PBS-0.3% Tween) (BD India Ink Reagent; Fisher Scientific, Waltham, MA, USA) and two washes in PBS. Membranes are allowed to air dry and images acquired with a ChemiDoc gel documentation system (Bio Rad). The membranes were subsequently dipped in 100% methanol solution and immediately blocked in 5% protease-free bovine serum albumin (BSA) for 1 h and incubated overnight at 4 °C with rabbit monoclonal anti-GLT-1 primary antibody (1:250,000 dilution; Abcam, Cambridge, United Kingdom, cat #247205). After washing with a solution containing 0.1% Tween-20 and TBS 1X (0.05 mol/L Tris, 0.15 mol/L NaCl), the membranes were incubated with anti-rabbit secondary IgG (1:10,000 dilution; Sigma-Aldrich cat #A9169) for 1 h at room temperature. Final detection was performed with enhanced chemiluminescence methodology (SuperSignal^®^ West Dura Extended Duration Substrate; Pierce, Rockford, IL, USA) and the intensity of the signal was measured using a ChemiDoc gel documentation system (Bio Rad). Detection was optimized to avoid saturation of the bands and linearity was tested prior to the selection of final detection parameters. Densitometry performed on the India Ink-stained membrane was used to correct for minor differences in protein loading. Values of each band were calculated with ImageLab Software (Bio-Rad), subtracting the background of the sample to the GLT-1 band signal (approximately 70 kDa). Densitometry analysis of the India Ink-stained membrane was performed to correct for minor differences in protein loading and used for normalization of the bands [33,34]. In some experiments we utilized the cerebellum to measure GLT-1 levels, whereas in others the cortex was used. The cerebellum was collected to quantify global GLT-1 upregulation by LDN in the same animals in which the experiments were performed and where the infarct sizes were measured in the cortical regions making them not suitable for Western blot. The cortex was used for GLT-1 measurements when TTC staining was not performed on the cortical brain tissue.

### 2.7. Statistical Analysis

Diagnostic for normality criteria was performed using the Shapiro–Francia estimator. Presence of outliers was verified via Grubbs’ test. Data distribution was confirmed using central tendency and dispersion measures. Evaluation of mean ranks differences in infarct dimension sizes between control vs. experimental groups was performed using non-parametric Mann-Whitney U tests. Motor performance in the Rung Ladder Walk was assessed via an independent t-test performed by frontal and back paws. Mean protein expression levels stratified by gender were assessed by an independent samples t-test. These tests were performed in tandem with the Levene’s equality of variances test. We performed a pre/post analysis, taking time as a factor using a Wilcoxon test approach to evaluate mean change. The significant level (α) was set to ≤0.05, except for the normality and equality of variances tests (*p* > 0.05). All tests were two-tailed. R v.3.6 (Team R: A language and environment for statistical computing) and IBM Statistical Package for Social Sciences (IBM-SPSS, Chicago, IL, USA) v.23.0 for Windows were used.

## 3. Results

### 3.1. Effect of LDN/OSU-0232120 Given 24 h before Focal Ischemia on Stroke Outcomes in Male and Female Mice

**Figure 2:**
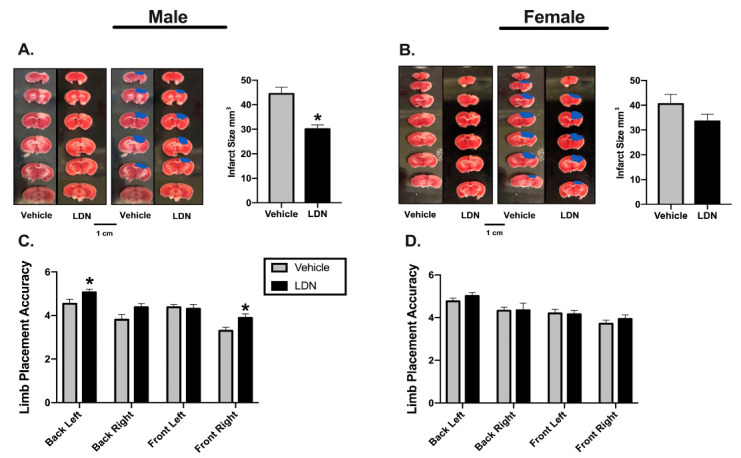
Effects of LDN 24 h before focal ischemia. Representative triphenyltetrazolium chloride (TTC) staining of coronal male (**A**) and female (**B**) brain sections (**left panels**). Infarcts are denoted in blue (**middle panels**) and a summary of the results are presented in the right panel (*n* = 9 for all groups). * indicates *p* < 0.05 using the Mann–Whitney U test. (**C**) Summary of male performance on the Rung Ladder 24 h after focal ischemia (*n* = 9 for both groups). (**D**) Summary of female performance on the Rung Ladder 24 h after focal ischemia (*n* = 9 for both groups). * indicates *p* < 0.05 using the Wilcoxon Signed Ranks Test.

Male or female mice (10–12 weeks-old) were injected (ip) with either LDN (40 kg/mg) or vehicle 24 h prior to inducing a focal ischemia in the sensorimotor cortex (Figure 1A). To assess the effect of LDN on stroke outcomes, we determined both sensorimotor performance of the mice using the Rung Ladder Walk (24 h after focal ischemia) as well as the infarct sizes (48 h after focal ischemia). Representative infarcts in vehicle and LDN-treated male mice are shown in Figure 2A where the lack of red color in the brain slices indicates the metabolically inactive portion of the slice where the infarct was induced. For better visualization, the infarcts are also colorized in blue. There was a statistically significant decrease in the infarct size of males treated with 40 mg/kg of LDN (30.0 ± 1.7 mm^3^; mean ± SEM; *n* = 9; *p* = 0.0004) 24 h prior to focal ischemia when compared with vehicle-treated males (44.4 ± 2.7 mm^3^; *n* = 9). Additionally, motor performance of males treated with 40 mg/kg of LDN 24 h after focal ischemia was significantly better for both the back-left paw (5.1 ± 0.2; *n* = 9; *p* = 0.030) and the front right paw (3.9 ± 0.2; *n* = 9; *p* = 0.016) in comparison to males treated with vehicle (back left 4.5 ± 0.2; *n* = 9 and front right 3.3 ± 0.2; *n* = 9) (Figure 2C).

In contrast, there was no significant difference in infarct size in 10–12-week-old female mice injected with either 40 mg/kg of LDN (33.5 ± 2.9 mm^3^; *n* = 9) or vehicle (40.5 ± 3.8 mm^3^; *n* = 9) when given 24 h prior to focal ischemia (Figure 2B). Furthermore, there was no difference observed on the rung ladder walk performance between females treated with 40 mg/kg LDN or vehicle (Figure 2D).

### 3.2. Effect of LDN/OSU-0232120 Given 2 h after Focal Ischemia on Stroke Outcomes in Male and Female Mice

**Figure 3:**
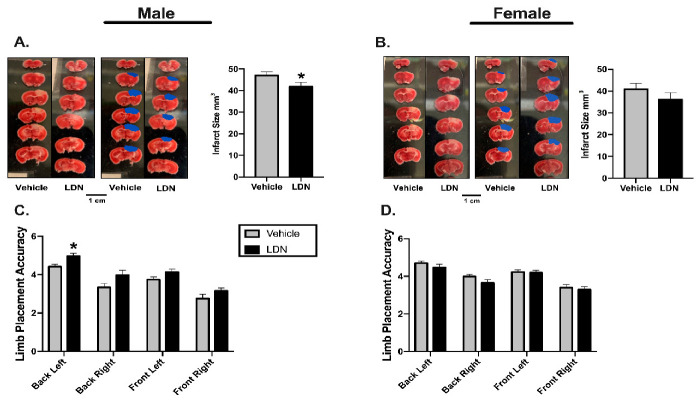
Effects of LDN 2 h after focal ischemia. Representative TTC staining of coronal male (**A**) and female (**B**) brain sections (**left panels**). Infarcts are denoted in blue (**middle panels**) and a summary of the results are presented in the right panel (*n* = 10 for all groups). (**C**) Summary of male performance on the Rung Ladder 24 h after focal ischemia (*n* = 10 for both groups) (**D**) Summary of female performance on the Rung Ladder 24 h after focal ischemia (*n* = 10 for both groups). * indicates *p* < 0.05 using the Wilcoxon Signed Ranks Test.

In order to assess the potential therapeutic application of LDN, we determined the effects of administering 40 mg/kg of LDN after focal ischemia on acute stroke outcomes (Figure 1B). To this end, male and female mice (10–12 weeks-old) were injected (ip) with either 40 mg/kg LDN or vehicle 2 h after inducing focal ischemia in the sensorimotor cortex. As above, we determined both sensorimotor performance of the mice (24 h after focal ischemia) as well as quantifying the infarct sizes (48 h after focal ischemia). Representative infarcts in brains of male and female mice are shown in Figure 3A,B, respectively. The infarct sizes of males treated with 40 mg/kg LDN two hours after focal ischemia were significantly smaller (41.9 ± 1.9 mm^3^; mean ± SEM; *n* = 10; *p* = 0.03) than those in brains of vehicle-treated male mice (47.1 ± 1.8 mm^3^; *n* = 10). In addition, males treated with 40 mg/kg LDN 2 h following the ischemic insult displayed better motor performance with their back-left paw (5.0 ± 0.2; *n* = 10; *p* = 0.002) compared to vehicle-treated male mice (4.4 ± 0.1; *n* = 10) (Figure 3A,C).

Similar to our previous findings (Figure 2B,D), injecting LDN 40 mg/kg 2 h after focal ischemia did not improve acute stroke outcomes in female mice (Figure 3B). There was no significant difference in the infarct size of LDN-treated female mice (36.1 ± 3.1 mm^3^; *n* = 10) when compared to vehicle-treated females (40.9 ± 2.8 mm^3^; *n* = 10). Moreover, there was no difference observed on the rung ladder walk performance between females treated with 40 mg/kg LDN or vehicle (Figure 3D).

### 3.3. Effect of LDN/OSU-0232120 on GLT-1 Protein Levels in Brains of Male and Female Mice

**Figure 4:**
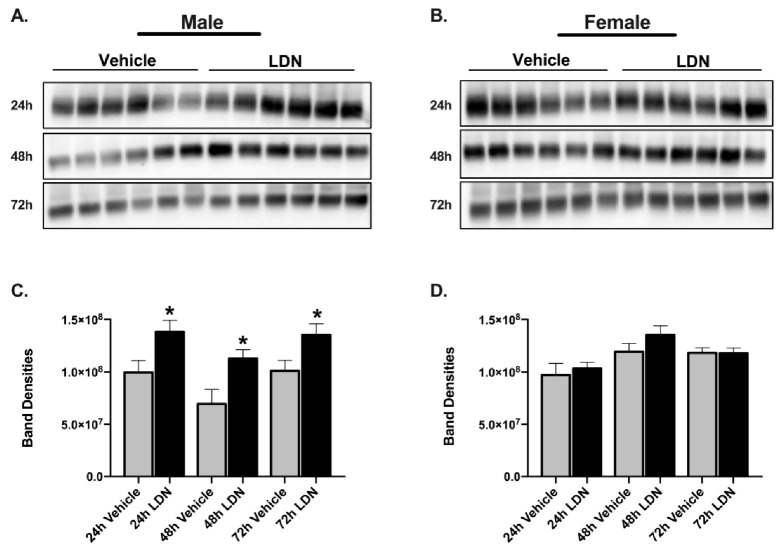
Expression of GLT-1 in brains after a single injection of vehicle or LDN-OSU 0232120 (LDN). (**A**) Western blots showing GLT-1 expression in male brain 24, 48, and 72 h after vehicle or LDN 40 mg/kg treatment (full length blots in Supplementary Figure S1a,c,e, respectively). (**B**) Western blots showing GLT-1 expression in female brain 24, 48, and 72 h after vehicle or LDN 40 mg/kg treatment (full length blots in Supplementary Figure S2a,c,e, respectively). For both males and females, cortex was used for the 24-h group, whereas cerebellum was used for the 48- and 72-h groups. The GLT-1 monomer is found at approximately 70 kDa. (**C**) Summary of GLT-1 monomer expression in males 24 (99.5 × 106 ± 11.4 × 106), 48 (69.5 × 106 ± 13.8 × 106), and 72 h (100.0 × 106 ± 10 × 106) after treatment with either vehicle or LDN 40 mg/kg. (**D**) Summary of GLT-1 monomer expression in female brain 24 (97.0 × 106 ± 11.1 × 106), 48 (11.9 × 107 ± 7.86 × 106), and 72 h (11.8 × 107 ± 4.8 × 106) after treatment with either vehicle or LDN 40 mg/kg. Data are expressed as mean ± SEM with an *n* = 6 for all groups. An unpaired t-test was performed to assess changes in GLT-1 expression at each time point. * indicates *p* < 0.05. These tests were performed in tandem with the Levene’s equality of variances test.

LDN is a translational activator of GLT-1. In order to assess if LDN increased GLT-1 protein levels in the mice that were used for the previous experiments, we collected the cerebellum region of the brain at the time we euthanized the mice and determined infarct size. The cerebellum was collected to quantify global GLT-1 upregulation by LDN in the same animals in which the experiments were performed and where the infarct sizes were measured in the cortical regions making them not suitable for Western blot. The 72-h time points shown in Figure 4 were obtained from the mice in which LDN was given 24 h prior to focal ischemia (Figure 1A and Figure 2) and the 48-h time points shown in Figure 4 were obtained from mice in which LDN was given 2 h after the focal ischemia (Figure 1B and Figure 3). Using Western blot, we determined protein levels of GLT-1 in the cerebellar region of the 10–12-week-old male and female mice and found that the levels of GLT-1 protein in male mouse brain were significantly increased 48 (*p* = 0.02) and 72 (*p* = 0.03) h after a single LDN injection as compared with vehicle treated controls (Figure 4C). Surprisingly, LDN-treatment (40 mg/kg; ip) did not significantly increase GLT-1 expression in cerebellum of female mice either 48 or 72 h after injection (Figure 4D).

In the first experiment, LDN was given 24 h prior to inducing the focal ischemia in the sensorimotor cortex of male and female mice. For this reason, we used Western blot to determine if GLT-1 was increased in the cortical region of cortex of male and female mice injected with LDN (40 mg/kg) as compared with vehicle-treated mice. Consistent with the results in cerebellum at 48 and 72 h, LDN increased GLT-1 in the cortical region of male (*p* = 0.03), but not female mice 24 h after injection (Figure 4). Full length blots are shown in Supplementary Figure S1 for males and Figure S2 for females.

**Figure 5:**
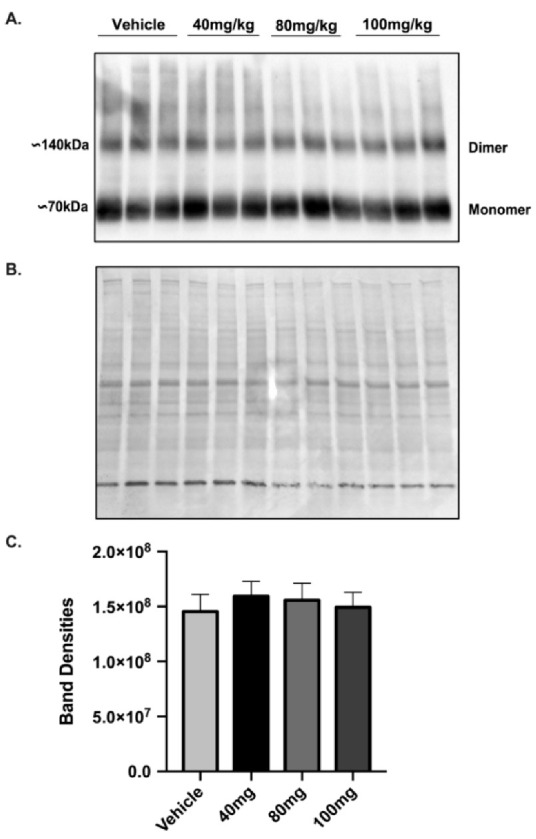
Expression of GLT-1 in female brain cortex after different doses of LDN-OSU 0232120 (LDN). (**A**) Western blot showing GLT-1 expression in the cortical region of female brains 24 h after vehicle or 40 mg/kg, 80 mg/kg and 100 mg/kg LDN (full length blot shown in Figure S3a). (**B**) India Ink staining of the membrane used for normalizing total protein loading (full length blot shown in Supplementary Figure S3b). (**C**) Summary of the Band Densities for all four conditions (after normalization for protein loading): vehicle (14.5 × 107 ± 1.5 × 107), 40 mg/kg LDN (15.9 × 107 ± 1.33 × 107), 80 mg/kg LDN (15.6 × 107 ± 1.5 × 107), 100 mg/kg LDN (14.9 × 107 ± 1.35 × 107). An ANOVA was performed to assess GLT-1 expression between groups. There was no significant difference on GLT-1 expression, *n* = 3 for all groups.

Because a single injection of 40 mg/kg LDN did not increase GLT-1 protein levels in brains of female mice at 24, 48 or 72 h after injection, we performed a dose response study to determine if higher doses of LDN could increase GLT-1 in cortex of female mice. We chose to examine GLT-1 levels at 24 h after injection of LDN because this was the time point of the maximal effect in males. We used cortical brain tissue for the assay because this is the region where focal ischemia was induced. In addition, we used concentrations of LDN up to 100 mg/kg since this concentration was shown to have no adverse effects on mice even when giving multiple treatments on subsequent days [21]. We found no difference in GLT-1 protein levels in cortices obtained from female mice treated with vehicle or 40–100 mg/kg of LDN (Figure 5). Full length blots are shown in Supplementary Figure S3.

## 4. Discussion

Astrocytes play an important role in reducing neuronal excitotoxicity and death induced by higher-than-normal extracellular glutamate concentrations [35]. They can serve as buffers for high glutamate concentrations in the brain as well as taking up extracellular glutamate; a process which is predominantly carried out by the glutamate transporter 1 (GLT-1) located in the astrocytic membrane [36]. During the last few years, the effects of increasing expression of GLT-1 have been studied in diseases such as epilepsy, Alzheimer’s disease, and traumatic brain injury [37,38,39]. Furthermore, GLT-1 has been studied as a potential therapeutic target in an assortment of neurological disorders [5]. Recently, the translational activation of GLT-1 and its potential therapeutic effect was studied in Amyotrophic Lateral Sclerosis and epilepsy [21]. Ischemic stroke shares a common pathophysiology with epilepsy: excitotoxicity, which promotes neuronal death during and after a stroke [40]. The main finding of the present study was that LDN can decrease infarct size in wild-type male mice and can improve their motor performance after focal ischemia. This improvement was not seen in wild-type female mice, thus suggesting that the therapeutic potential of LDN could only be for treating the physiological repercussions of an ischemic stroke in males.

Previously, Kong et al. [21] have shown that LDN delayed motor dysfunction in transgenic male and female mice expressing familiar ALS-linked mutant superoxide dismutase (SOD1) and improved the phenotype of a pilocarpine model of epilepsy in male mice [21]. In contrast to the wild-type mice used for the current study, the SOD1 mice model used to study the effects of LDN by Kong et al. [21] had substantial downregulation of GLT-1 expression as a direct consequence of the ALS pathology. In our study, we aimed to address the therapeutic potential of LDN as a treatment for ischemic stroke. LDN upregulates GLT-1 levels at a post-transcriptional level. It does not increase mRNA levels but acts as a polyribosome recruiter through Y-binding protein, thus facilitating synthesis of GLT-1 protein [21]. Following published LDN pharmacokinetics [21], GLT-1 levels were maximally increased 24 h after a single injection. Therefore, in the first experiment, we injected mice with LDN 24 h before inducing a focal ischemia with the purpose of having maximal expression of GLT-1 at the time of the surgery. In young adult males given LDN 24 h prior to photothrombosis, infarct sizes were smaller than male littermates treated with vehicle only. In contrast, females that were treated with LDN 24 h prior to inducing focal ischemia did not display any differences in infarct size when compared to vehicle-treated females.

Stroke is known as a cause of disability and motor dysfunction. This is why it is important to consider treatments that not only address the neuronal death associated with stroke, but also improve motor outcomes after stroke [41,42]. Males that were injected with LDN displayed better motor performance than males that received vehicle only as expected from the infarct size measurements. In contrast to the aforementioned findings, pre-treatment with LDN did not improve motor performance in female mice after focal ischemia.

Currently, the only non-invasive FDA approved treatment available for people suffering from strokes is tissue plasminogen activator (tPA). tPA has a narrow time window for administering the treatment which limits the amount of people who receive it [43]. It is estimated that only around 10% of patients suffering from stroke meet the criteria for tPA treatment [44]. Because of the limited treatment options patients have after suffering from a stroke, the fast upregulation of GLT-1 after injection with LDN [21] and considering our findings of a preventive LDN treatment for stroke, we performed experiments to determine if LDN given after focal ischemia could have beneficial outcomes. Male mice treated with LDN 2 h after focal ischemia displayed smaller infarct sizes and better motor performance than males treated with vehicle only. These studies examined a single injection at a single time point. Future studies could address the possibility of how the beneficial outcomes of LDN could be extended testing multiple time points and assessing how long and how often treatment with LDN can be beneficial after stroke.

On the other hand, females treated 2 h after focal ischemia with 40 mg/kg of LDN did not display differences in infarct sizes or motor performance in comparison with females that were treated with vehicle only. This difference between males and females can perhaps be explained by the apparent inability of LDN in concentrations up to 100 mg/kg to increase GLT-1 protein levels in cortex of 10–12-week-old C57BL/6NCrl female mice. It was previously published that a single injection of 40 mg/kg LDN had a sustained effect on the expression of GLT-1 in brains of male mice for 72 h [21]. Our results in male mice are consistent with previous findings, in that GLT-1 levels were increased in brain 24, 48, and 72 h after a single injection of 40 mg/kg of LDN. In comparison, 40 mg/kg of LDN did not increase GLT-1 expression at any time point in female mouse brain. Because there may be differences in the pharmacodynamics and pharmacokinetics of LDN in females and males, we increased the concentration delivered to females to assess the possibility that a higher dose of LDN was needed in females to increase GLT-1 expression [45]. We administered up to 100 mg/kg as the maximal dose because previous studies showed no adverse effects in mice in vivo [21]. Surprisingly, GLT-1 expression levels in cortex were not increased in female mice 24 h after a single injection of 100 mg/kg LDN as compared to vehicle-treated females.

GLT-1 has been studied as a potential therapeutic target for stroke, but most of the studies have utilized males only or cell culture systems that do not necessarily take into consideration the microenvironment each sex provides. Harvey and colleagues [18] increased GLT-1 expression in the area of subsequent infarction by injection of an adeno-associated viral vector expressing GLT-1 cDNA (AAV-GLT1) into cortical regions of male rats. This was done three weeks before performing MCAo and resulted in a decrease in the duration and magnitude of extracellular glutamate release during MCAo. Moreover, the injection of AAV-GLT1 resulted in significant improvement in behavioral recovery after stroke compared to control animals receiving an adeno-associated viral vector expressing GFP [18]. Hu and colleagues [19] used ceftriaxone, an antibiotic which transcriptionally upregulates GLT-1 protein, to attempt to improve outcomes of global ischemia in male rats. They found that administration of ceftriaxone increased GLT-1 expression and glutamate uptake in CA1 hippocampus. Furthermore, ceftriaxone reduced delayed neuronal death in CA1 hippocampus in males treated with ceftriaxone when given either before or after global ischemia [19].

The previous studies involve experiments performed in male rats, which leads to the question: can GLT-1 be upregulated in females? What has been shown is that LDN can upregulate GLT-1 levels in the female adult superoxide dismutase (SOD1) (G93A) transgenic mouse model of ALS [21]. This ALS model is characterized by a loss of motor neurons and a downregulation in spinal cord GLT-1 levels at approximately 80–90 days of age. Daily injections of LDN (ip, 40 mg/kg) starting at 84 days of age until death restored GLT-1 in the spinal cord of female SOD1 mice to levels comparable to those in wildtype female mice and significantly delayed motor function decline and prolonged overall survival [21]. This indicates that LDN can increase GLT-1 levels in female SOD1 mice at a time when GLT-1 levels are dramatically reduced as the disease progresses. Nevertheless, the authors did not demonstrate if LDN can modulate GLT-1 levels in wild type female mice [21], a question we aimed to answer in this paper.

There are known sex-specific differences in stroke outcomes between males and females. Premenopausal women have fewer strokes than similarly aged males, but postmenopausal women have more strokes than males and the outcomes of these strokes are more severe. In premenopausal women, estrogen has a neuroprotective effect which involves various cellular mechanisms including vascular reactivity [46], reducing oxidative stress [47], and reduction of fatty plaques in the arteries [48], resulting in smaller infarct sizes compared to males [49].

Estradiol with the involvement of transforming growth factor-α (TGF-α) can upregulate GLT-1 expression and function in rat neonatal cultured astrocytes [50]. Furthermore, tamoxifen (a selective estrogen receptor modulator) can upregulate GLT-1 levels through a mechanism involving phosphorylation of CREB and recruitment of CREB and the NFкβ subunits p65 and p50 to the GLT-1 promoter [51]. Nevertheless, these findings were performed in cultured astrocytes and to date there are no studies showing that estrogen increases GLT-1 expression in wildtype mice in vivo. The mechanism by which LDN upregulates GLT-1 is through the phosphorylation and activation of PKC, probably PKC-1, which subsequently activates the Y-box-binding protein 1 (YB-1) [21]. YB-1 binds to the mRNA and promotes translation of GLT-1 [21]. The present study was performed in young female mice where it is known the estrogen levels are higher in comparison to young male mice [52]. Interestingly, we found similar levels of GLT-1 expression between vehicle-treated male and female mice (Figure 4) suggesting that there are differences in GLT-1 regulation in vivo and in vitro. Moreover, treatment with LDN upregulated GLT-1 expression levels in males, but not in females.

Beyond the previously described hormonal sex differences, epigenetic factors can potentially contribute to the difference observed in LDN modulation in male and female mice. The genetic chromosomal component of males and females is different and associations with either X or Y chromosomes have been hypothesized to mediate the diverse physiological response of males and females to ischemic stroke [53,54]. Epigenetic mechanisms such as DNA methylation and demethylation play a role in gene expression and protein expression that affect neuroprotection pathways in both males and females [55,56,57]. Thus, epigenetic differences between males and females could potentially have a role in the failure of LDN to upregulate GLT-1 levels in females.

In summary, our studies demonstrate that LDN has a beneficial effect on stroke outcomes (reducing infarct size and associated motor impairment) in young adult male, but not young wild-type female mice. The results obtained in this study open the question of how to target treatments based on sex differences and to take into account the physiological environment each sex displays. The beneficial effects observed in males treated with LDN before and after the ischemic event, demonstrate the therapeutic potential translational modulators possess for pathologies such as ischemic strokes.

## 5. Conclusions

In these studies, we have shown a potential therapeutic for ischemic stroke. We have demonstrated how upregulating levels of GLT-1 with LDN/OSU 0212320 results in smaller infarct size as well as better motor performance in male mice. One intriguing finding was the sex-specific difference in response to LDN treatment. Females treated with LDN before or after focal ischemia did not display the same beneficial effects observed in males. These findings expand the potential targets and therapeutics for improving negative outcomes of ischemic events.

## Data Availability

Data are contained within the article or supplementary material.

## Supplementary Materials

The following are available online at https://www.mdpi.com/2076-3425/11/1/76/s1, Figure S1: Expression of GLT-1 in male brains after a single injection of vehicle or LDN-OSU 0232120 (LDN); Figure S2: Expression of GLT-1 in female brains after a single injection of vehicle or LDN-OSU 0232120 (LDN); Figure S3: Expression of GLT-1 in female brain cortex after different doses of LDN-OSU 0232120 (LDN).
