# Erratum: Tejeda-Bayron et al. Activation of Glutamate Transporter-1 (GLT-1) Confers Sex-Dependent Neuroprotection in Brain Ischemia. *Brain Sci.* 2021, *11*, 76

**DOI:** 10.3390/brainsci11091144

**Published:** 2021-08-29

**Authors:** Flavia A. Tejeda-Bayron, David E. Rivera-Aponte, Christian J. Malpica-Nieves, Gerónimo Maldonado-Martínez, Héctor M. Maldonado, Serguei N. Skatchkov, Misty J. Eaton

**Affiliations:** 1 Department of Biochemistry, School of Medicine, Universidad Central del Caribe, Bayamón, PR 00960-6032, USA; 415ftejeda@uccaribe.edu (F.A.T.-B.); david.rivera@uccaribe.edu (D.E.R.-A.); 415cmalpica@uccaribe.edu (C.J.M.-N.); 2 School of Chiropractic, Universidad Central del Caribe, Bayamón, PR 00960-6032, USA; geronimo.maldonado@gmail.com; 3 Biology Department, University of Puerto Rico—Río Piedras Campus, Río Piedras, PR 00924-2537, USA; 4 Department of Pharmacology, School of Medicine, Universidad Central del Caribe, Bayamón, PR 00960-6032, USA; hmaldonado1@gmail.com; 5 Department of Physiology and Biochemistry, School of Medicine, Universidad Central del Caribe, Bayamón, PR 00960-6032, USA; serguei.skatchkov@uccaribe.edu

The authors wish to make the following corrections to this paper [1]. There are typos in the numbers associated with the compound LDN/OSU 0212320. The numbers were incorrectly typed as LDN/OSU 0232120 in some areas of the manuscript and Supplementary Materials. The correct information is:

In the fifth paragraph of Introduction:

In 2014, Kong and colleagues characterized a compound, LDN/OSU 0212320 (LDN), that upregulated GLT-1 protein levels at the post-transcriptional level as fast as two hours after injection.

In the sub-title of Results:

*3.1. Effect of LDN/OSU 0212320 Given 24 h before Focal Ischemia on Stroke Outcomes in Male and Female Mice*

*3.2. Effect of LDN/OSU 0212320 Given 2 h after Focal Ischemia on Stroke Outcomes in Male and Female Mice*

*3.3. Effect of LDN/OSU 0212320 on GLT-1 Protein Levels in Brains of Male and Female Mice*

In Figure 4 caption:

Expression of GLT-1 in brains after a single injection of vehicle or LDN/OSU 0212320 (LDN).

In Figure 5 caption:

Expression of GLT-1 in female brain cortex after different doses of LDN/OSU 0212320 (LDN).

In the Supplementary Materials: 

The following are available online at www.mdpi.com/2076-3425/11/1/76/s1, Figure S1: Expression of GLT-1 in male brains after a single injection of vehicle or LDN/OSU 0212320 (LDN); Figure S2: Expression of GLT-1 in female brains after a single injection of vehicle or LDN/OSU 0212320 (LDN); Figure S3: Expression of GLT-1 in female brain cortex after different doses of LDN/OSU 0212320 (LDN).

In the supplementary file, the figure caption should be:

Supplemental Figure S1: Expression of GLT-1 in male brains after a single injection of vehicle or LDN/OSU 0212320 (LDN).

Supplemental Figure S2: Expression of GLT-1 in female brains after a single injection of vehicle or LDN/OSU 0212320 (LDN).

Supplemental Figure S3: Expression of GLT-1 in female brain cortex after different doses of LDN/OSU 0212320 (LDN).

The authors would like to apologize for any inconvenience caused to the readers by these changes.
