# Supplementary material for: Activation of Glutamate Transporter-1 (GLT-1) Confers Sex-Dependent Neuroprotection in Brain Ischemia
Source: Brain Sci. 2021 Jan 8;11(1):76. doi: 10.3390/brainsci11010076 (PMC7827447; doi:10.3390/brainsci11010076)
Supplement: Supplementary file 1 [file brainsci-11-00076-s001.pdf]

Activation of Glutamate Transporter-1 (GLT-1) Confers Sex-Dependent Neuroprotection in Brain Ischemia

Flavia A. Tejeda-Bayron [1], David E. Rivera-Aponte [2], Christian J. Malpica-Nieves [3], Gerónimo Maldonado-Martínez [4], Héctor M. Maldonado [5], Serguei N. Skatchkov [6] and Misty J. Eaton [7*]

[1] Department of Biochemistry School of Medicine Universidad Central del Caribe; 415ftejeda@uccaribe.edu
[2] Department of Biochemistry School of Medicine Universidad Central del Caribe; david.rivera@uccaribe.edu
[3] Department of Biochemistry School of Medicine Universidad Central del Caribe; 415cmalpica@uccaribe.edu
[4] School of Chiropractor Universidad Central del Caribe; geronimo.maldonado@gmail.com
[5] Department of Pharmacology School of Medicine Universidad Central del Caribe; hmaldonado1@gmail.com
[6] Department of Physiology School of Medicine Universidad Central del Caribe; serguei.skatchkov@uccaribe.edu
[7] Department of Biochemistry School of Medicine Universidad Central del Caribe; misty.eaton@uccaribe.edu

Supplementary Figures

a.

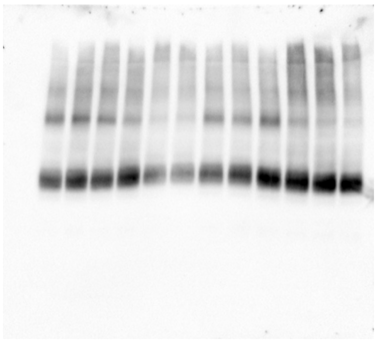

b.

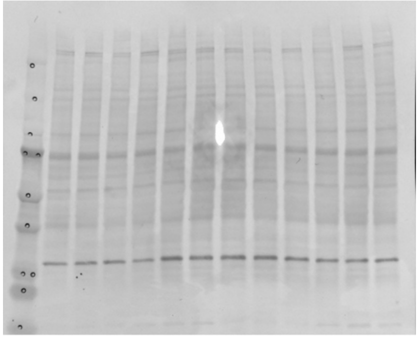

c.

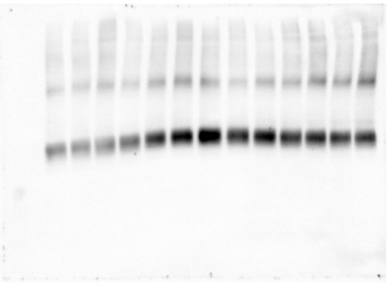

d.

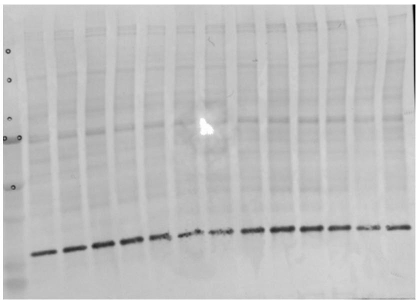

e.

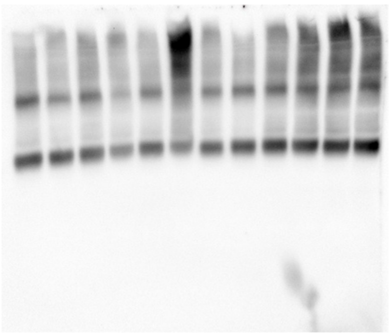

f.

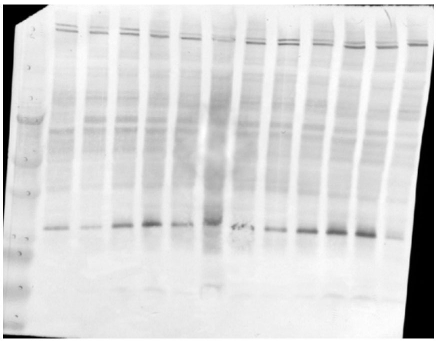

Supplemental Figure S1: Expression of GLT-1 in male brains after a single injection of vehicle or LDN-OSU 0232120 (LDN). Full length Western blots showing GLT-1 expression in male brain 24 (**a**), 48 (**c**) and 72 (**e**) hours after vehicle or LDN 40 mg/kg treatment. India Ink staining showing total protein

concentration for Western Blots performed in male brain 24 (**b**), 48 (**d**), and 72 (**f**) hours after vehicle or LDN 40mg/kg treatment.

a.

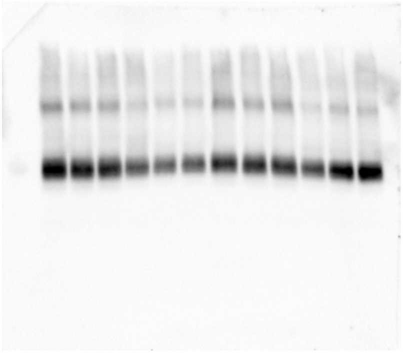

b.

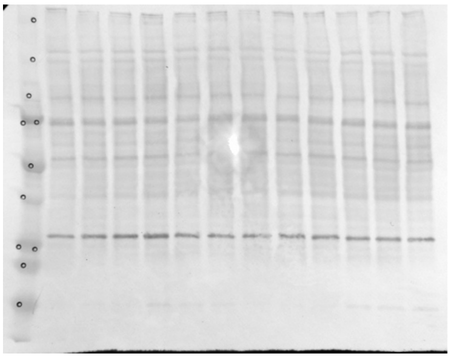

c.

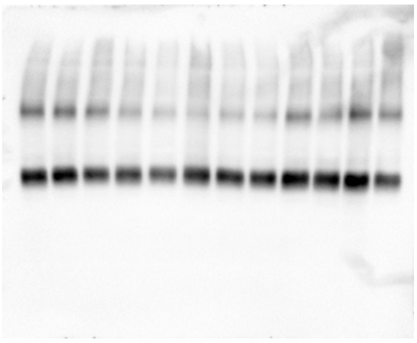

d.

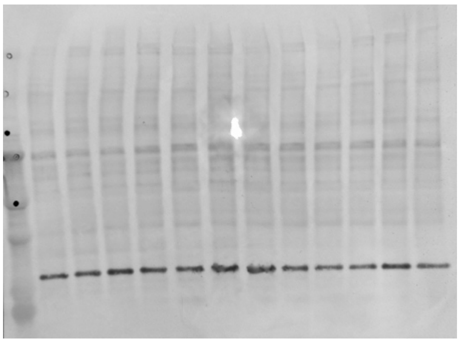

e.

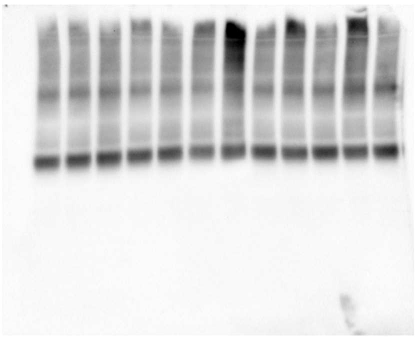

f.

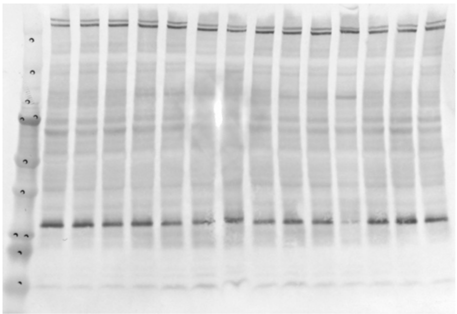

Supplemental Figure S2: Expression of GLT-1 in female brains after a single injection of vehicle or LDN-OSU 0232120 (LDN). Full length Western blots showing GLT-1 expression in female brain 24 (**a**),

48 (**c**) and 72 (**e**) hours after vehicle or LDN 40 mg/kg treatment. India Ink staining showing total protein concentration for Western Blots performed in female brain 24 (**b**), 48 (**d**), and 72 (**f**) hours after vehicle or LDN 40mg/kg treatment.

a.

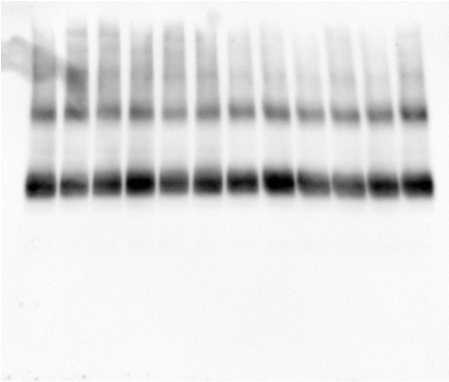

b.

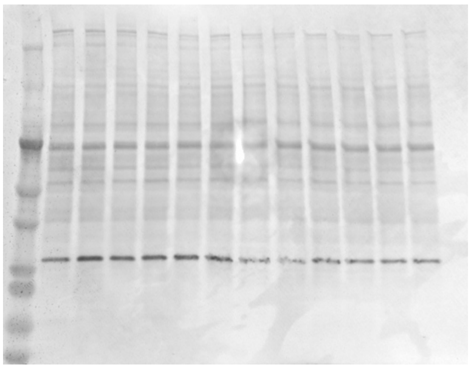

Supplemental Figure S3: Expression of GLT-1 in female brain cortex after different doses of LDN-OSU 0232120 (LDN). (a) Full length Western blot showing GLT-1 expression in the cortical region of female brains 24 hours after vehicle or 40 mg/kg, 80 mg/kg and 100 mg/kg LDN. (**b)** India Ink staining showing total protein concentration for Western Blot performed in female brain cortex after different doses of LDN-OSU 0232120 (LDN).
